# Damaged DNA-binding protein 2 (DDB2) protects against UV irradiation in human cells and Drosophila

**DOI:** 10.1186/1423-0127-17-27

**Published:** 2010-04-17

**Authors:** Nian-Kang Sun, Chun-Ling Sun, Chia-Hua Lin, Li-Mai Pai, Chuck CK Chao

**Affiliations:** 1 Department of Biochemistry and Molecular Biology, Chang Gung University, Gueishan, Taoyuan 333, Taiwan; 2 Division of Biomedical Sciences, Chang Gung Institute of Technology, Gueishan, Taoyuan 333, Taiwan

## Abstract

**Background:**

We observed previously that cisplatin-resistant HeLa cells were cross-resistant to UV light due to accumulation of DDB2, a protein implicated in DNA repair. More recently, we found that cFLIP, which represents an anti-apoptotic protein whose level is induced by DDB2, was implicated in preventing apoptosis induced by death-receptor signaling. In the present study, we investigated whether DDB2 has a protective role against UV irradiation and whether cFLIP is also involved in this process.

**Methods:**

We explored the role of DDB2 in mediating UV resistance in both human cells and Drosophila. To do so, DDB2 was overexpressed by using a full-length open reading frame cDNA. Conversely, DDB2 and cFLIP were suppressed by using antisense oligonucleotides. Cell survival was measured using a colony forming assay. Apoptosis was monitored by examination of nuclear morphology, as well as by flow cytometry and Western blot analyses. A transcription reporter assay was also used to assess transcription of cFLIP.

**Results:**

We first observed that the cFLIP protein was upregulated in UV-resistant HeLa cells. In addition, the cFLIP protein could be induced by stable expression of DDB2 in these cells. Notably, the anti-apoptotic effect of DDB2 against UV irradiation was largely attenuated by knockdown of cFLIP with antisense oligonucleotides in HeLa cells. Moreover, overexpression of DDB2 did not protect against UV in VA13 and XP-A cell lines which both lack cFLIP. Interestingly, ectopic expression of human DDB2 in *Drosophila *dramatically inhibited UV-induced fly death compared to control GFP expression. On the other hand, expression of DDB2 failed to rescue a different type of apoptosis induced by the genes *Reaper *or *eiger*.

**Conclusion:**

Our results show that DDB2 protects against UV stress in a cFLIP-dependent manner. In addition, the protective role of DDB2 against UV irradiation was found to be conserved in divergent living organisms such as human and *Drosophila*. In addition, UV irradiation may activate a cFLIP-regulated apoptotic pathway in certain cells.

## Background

Apoptosis plays an important role during the development and the lifespan of multicellular organisms by eliminating various cells via processes mediated mainly by caspase enzymes [1,2]. DNA-damaging agents such as ultraviolet (UV) light or chemotherapeutic agents can cause apoptosis via pathways that involve mitochondria [3,4]. Several intracellular signals are known to regulate the apoptosis process. For instance, Bcl-2 and Bcl-xL inhibit apoptosis by preventing the mitochondrial changes that lead to activation of the Apaf-1/caspase-9-apoptosome [5]. In addition, the X-linked inhibitor of apoptosis proteins (XIAP) prevents apoptosis by directly inhibiting the action of caspases [6]. On the other hand, apoptosis can be activated by death receptors, which are part of the superfamily of tumor necrosis factor (TNF) receptor, such as Fas, which recruits caspase-8 via the FADD/MORT1 adaptor [7,8].

The Fas signaling pathway is a complex set of events that can be regulated by both cellular and viral proteins, including cellular-FLICE inhibitory proteins (cFLIP) [9]. A recent study showed that UV irradiation could cause apoptosis by activating the Fas signaling pathway in various cells [10]. The cFLIP associates with the signaling complex which is downstream of death receptors. Three cFLIP splicing variants have been identified: cFLIPL, cFLIPS, and cFLIPR, and all three have been shown to act as inhibitors of apoptosis. cFLIP is a catalytically inactive homologue of pro-caspase-8/10 that negatively interferes with pro-apoptotic signaling. The importance of cFLIP in humans was shown by the finding that dysregulation of cFLIP expression is observed in numerous autoimmune diseases and cancers [11].

UV light is known to induce DNA repair in irradiated cells. Damaged DNA-binding (DDB) proteins, which mediate a key process in nucleotide excision repair after UV damage, represent a complex consisting of the two subunits DDB1 (127 kDa) and DDB2 (48 kDa) [12,13]. The human DDB2 cDNA has been characterized [14] and the DDB2 protein has been demonstrated to rely on DDB1 to recognize UV-damaged DNA [15]. We and others have previously found that overexpression of DDB2 enhances nuclear excision repair in both hamster [16,17] and human cells [18,19]. Notably, cisplatin-resistant HeLa cells are cross-resistant to UV, and exhibit both stronger DNA repair processes and increased DDB activity compared to parental cells [20,21]. We also found that the cellular level of DDB2 protein may regulate sensitivity to UV irradiation [22]. However, the complete mechanism underlying resistance to UV irradiation still remains unclear. Recently, we demonstrated that overexpression of DDB2 induced cFLIP, and partially inhibited TNF-induced apoptosis [23], an observation which may be related to UV resistance.

In the present study, DDB2 was found to increase resistance to UV in HeLa cells in a cFLIP-dependent manner. Notably, ectopic expression of human DDB2 in *Drosophila *also protected this organism against UV irradiation. These results support the notion that DDB2-mediated DNA repair may be required in UV resistance. In addition, UV irradiation may activate a cFLIP-regulated apoptotic pathway in certain cells.

## Methods

### Cell lines and culture

Human HeLa (S3), VA13, XP-A, and HEK293 cells (obtained from the American Type Tissue Collection), and cisplatin-resistant HeLa cell lines (HR3 and HR18) [21,22] were prepared and maintained as described previously [22]. HR18, a DDB2 knockdown cell line, was obtained by transfecting DDB2 antisense cDNA in HR3 cells [22].

### Western blot analysis

Cell extracts and Western blot analysis were done as previously described [22]. Fifty μg of proteins were detected with antibodies reactive against DDB2 [22], caspase-8, caspase-9 (Cell Signaling Technology, Beverly, MA), caspase-3, PARP, DFF, cFLIP, Bcl-2, Bcl-xL, or β-actin (Santa Cruz Biotechnology, Santa Cruz, CA). The antigen-antibody complexes were visualized using enhanced chemiluminescence reaction according to the instructions provided by the manufacturer (Pierce, Rockford, IL).

### Cell clonogenicity and apoptosis

Sensitivity to UV irradiation (UVC, 0.5 J/m^2^/s) was determined by clonogenicity and apoptosis as described previously [22]. In some cases, HR18 cells were either infected with β-Gal or DDB2 recombinant virus for 36 hrs prior to exposure to UV. For assessment of clonogenicity, a colony with at least 50 cells was used. For assessment of apoptotic cells [23], at least 500 nuclei were examined for each sample 24 hrs after UV irradiation as described previously [22]. Apoptotic cells were also determined from the distribution of sub-G1 cells by using flow cytometry [24]. Stained nuclei were then analyzed using a Becton Dickinson FACScan (San Jose, CA) with 10,000 events per determination. LYSYS II software was used to assess cell cycle distribution.

### Construction and production of recombinant DDB2 adenovirus

Replication-deficient recombinant adenoviruses containing either DDB2 or β-Gal were generated as described earlier [22,25]. Cells were infected with adenoviruses at a multiplicity of infection (MOI) of 3,000 for 36 hrs unless indicated otherwise before UV irradiation.

### Real-Time polymerase chain reaction (RT-PCR)

Real-time PCR was performed using an ABI Prism 7700 (Applied Biosystems, Foster City, CA) as described before [26]. Total RNA (10 μg) was converted into cDNA using oligo-dT primers with the SuperScript first-strand synthesis system (Invitrogen, Carlsbad, CA). PCR amplifications of 10 ng of the cDNA were performed in triplicates using Taq-Man Master Mix (Applied Biosystems). Quantification and fold change of RNA abundance was calculated using the standard curve method. GenBank sequence numbers (U97074, U18300, NM_000996) were used to design primers for cFLIP, DDB2, and ribosomal protein L35a, respectively [26].

### Inhibition assay with antisense oligonucleotides (ASO)

For antisense experiments, phosphothioated cFLIP antisense oligonucleotides (ASO) (ACTTGTCCCTGCTCCTTGAA) or control phosphothioated oligonucleotides (GGATGGTCCCCCCTCCACCAGGAGA), which were synthesized by PAN Facility, Stanford University, were delivered into cells by lipofection (Invitrogen) at a final concentration of 600 nM, as described earlier [26]. After 4 hrs, the medium was removed, and was replaced with the appropriate cell growth medium containing the oligonucleotides for 24 hrs. For the experiments requiring overexpression of DDB2 or β-Gal, cells were also replaced with growth medium containing the respective viruses (Ad-DDB2 or Ad-β-Gal) for 36 hrs, and then the cells were stimulated with UV and incubated for an additional 24 hrs.

### Isolation and expression of cFLIP cDNA

Human cFLIP cDNA (The GenBank sequence number U97074) containing full-length open reading frame was isolated by PCR amplification of total RNA extract (2 μg) from HeLa cells. The following PCR primer sequences were used: 5' **GGTACC**GACCCTTGTGAGCTTCCCTAGTCTAAG 3' (forward primer) and 5'**CTCGAG**GGTGTGAGCCACTACGCCCAG (reverse primer), with both containing extra sequences for the restriction sites *Kpn *I and *Xho *I (bold), respectively. The PCR products were ligated into the pGEM-Teasy vector (Promega, Madison, WI), and then subcloned into the expression vector pcDNA3 (Invitrogen) by using the enzymes *Kpn *I and *Xho *I, to obtained pcDNA3-cFLIP. The isolated cDNA sequence was confirmed by automatic sequencing, and expressed in HEK293 cells by transfection with lipofectamine (Invitrogen).

### cFLIP promoter reporter assay

Human cFLIP promoter fused to luciferase reporter gene was applied to study the transactivation by DDB2. Sub-confluent growing cells were co-transfected with a total of 3 μg of plasmid DNA containing 1 μg of pFP-1, with a potential cFLIP promoter region (flanking from -920 to +43 of cFLIP exon 1 start site; GenBank accession number AF238465, a kind gift from Dr. B. M. Evers, The University of Texas Medical Branch at Galveston, Galveston, TX), or its deletion variants, together with the indicated amount of pcDNA3, pcDNA3-DDB2, or pcDNA3-HMG1. The deletion variants of cFLIP promoter were generated by manipulating pFP-1 with the appropriate restriction endonucleases and ligases. After incubation for the time indicated, the cells were lysed, and the luciferase activity of the lysates was measured (Promega) with a β-scintillation counter (PerkinElmer, Waltham, MA).

### Overexpression of DDB2 in Drosophila and measurement of toxicity

The enzymes *Bgl*II/*Not*I were used to release GFP and hDDB2 from pEGFP-N1 and pEGFP-N1-hDDB2. The resulting fragments were subcloned into the expression vector pUAST. The constructs were named pUG and pUG-hDDB2, respectively. Ubiquitous expression of GFP and GFP-hDDB2 were driven by hsGal4. After 2 hrs of heat shock, dechorinated embryos or dissected larvae were homogenized in 2× sample buffer.

### *Survival assay *in Drosophila

The third instar larvae were heat-shocked at 37°C for 2 hrs to induce GFP or GFP-hDDB2 expression, followed by exposure to UV at 0-80 J/m^2^. Fifty larvae were exposed to each dose. Two independent insertion lines of each construct and four independent experiments were carried out. After exposure to UV, the larvae were incubated at 25°C until the adult flies eclosed.

### *Apoptosis assay *in fruit flies

The following transgenic fly strains were used for the genetic analysis: *GMR>Reaper *(or *GMR>Rpr*), *UAST-DDB2*, *UAST-p35*, *GMR-Gal4*, *UAST-eiger *were used. As a control, p35 (baculovirus cell death inhibitor) was used to block reaper-induced apoptosis in *Drosophila*. All genetic crosses were performed at either 25°C or 29°C. The strain *GMR>Reaper *features a rough and reduced eye phenotype. The degree of apoptosis was determined by eye phenotype.

## Results

### Resistance to UV in cisplatin-resistant HeLa cells is associated with increased levels of DDB2

**Figure 1:**
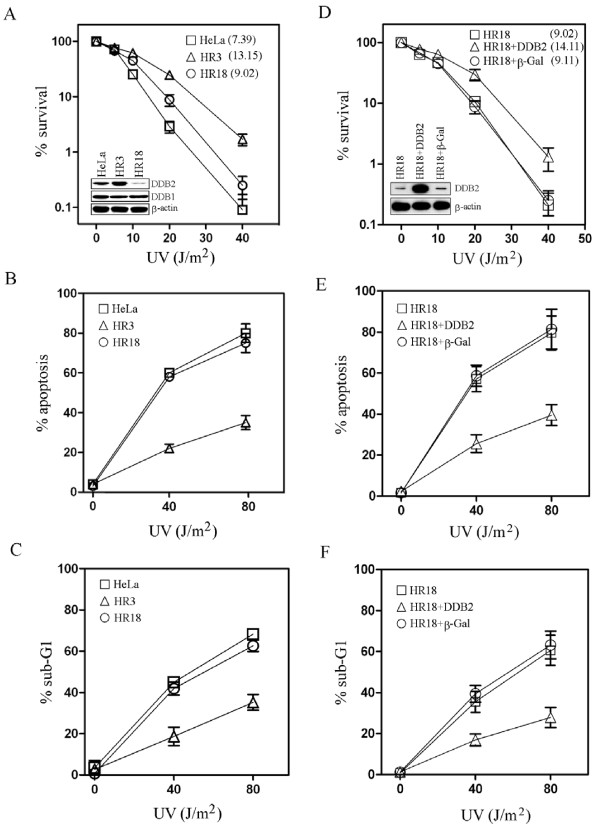
**Protection against UV-induced cytotoxicity by forced expression of DDB2**. (A, B, C) Sensitivity to UV is presented for stable cell lines indicated or for (D, E, F) HR18 cells which express DDB2. The protein levels of DDB2, DDB1, and β-actin are shown in the insets. The plotted values represent means ± S.D. of experiments performed in triplicates. IC_50 _values were also indicated in A and D.

We first assessed the level of DDB2 protein in the cisplatin-resistant HeLa cells HR3 and HR18. While HR3 cells were obtained by treating HeLa cells with repeated cycles of cisplatin, HR18 were derived from HR3 cells following expression of antisense cDNA to knockdown DDB2 [22]. By western blot analysis, we observed that the amount of DDB2 protein in HR3 cells was around two times that seen in control HeLa cells (Fig. 1A). On the other hand, DDB2 in HR18 cells was lower than in control HeLa cells (Fig. 1A). When the viability of these cells upon UV irradiation was monitored, we noted that HR3 cells were more resistant to UV than HR18 or control HeLa cells (Fig. 1A). The level of DDB1 in HR3 and HR18 was similar to control cells (Fig. 1A), an observation which suggested that resistance to UV in these cells may be associated mostly with DDB2. Next, we measured the level of apoptosis in the UV-irradiated cells by assessing nuclear morphology (Fig. 1B) or by flow cytometry (Fig. 1C). We confirmed that HR3 cells were more resistant to UV than HR18 or control HeLa cells in both assays (Fig. 1B and 1C). Overexpression of DDB2 in HR18 cells was shown to increase resistance to UV in these cells compared to overexpression of β-Gal or to control HR18 cells (Fig. 1D). In addition, HR18 cells overexpressing DDB2 showed lower apoptosis in response to UV compared to control cells (Fig. 1E and 1F). These results indicate that resistance to UV is associated with an increased level of DDB2.

### DDB2 protects against UV-induced apoptosis in a caspase-8 and/or caspase-9 dependent manner

**Figure 2:**
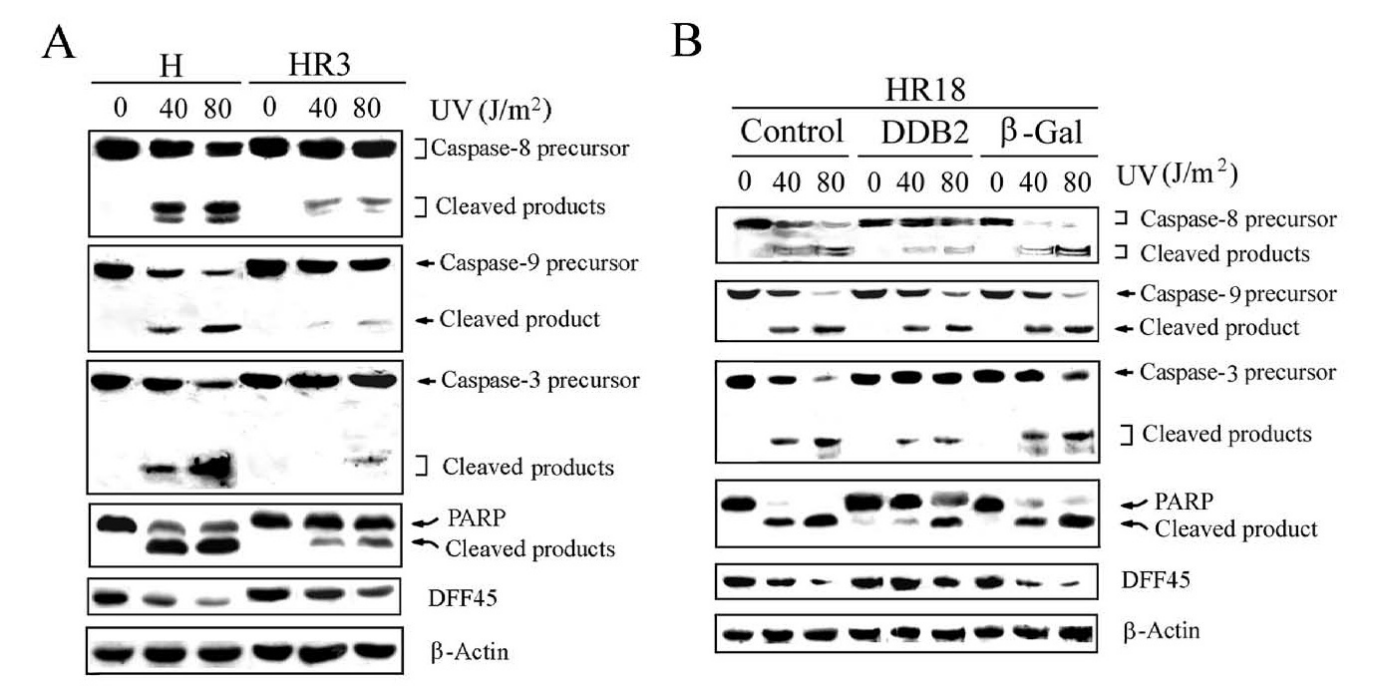
**Overexpression of DDB2 is associated with reduced caspase activation following UV treatment**. (A) Reduced caspase activation in cisplatin-resistant cells following UV irradiation. Cell extracts were immunoblotted with the antibodies indicated. (B) Reduced caspase activation in DDB2-expressing cells following UV irradiation.

We further investigated the levels of apoptotic markers in these cells. While UV irradiation was found to induce the cleavage and activation of caspases-8, 9, and 3 in both control HeLa and HR3 cells, the cleavage/activation was considerably reduced in HR3 cells (Fig. 2A). Similarly, cleavage of both PARP and DNA fragmentation factor (DFF45) substrates was also decreased in UV-irradiated HR3 cells compared to control cells (Fig. 1A). In UV-irradiated HR18 cells, overexpression of DDB2 was found to decrease the cleavage/activation of caspases-8, 9, and 3 compared to control cells (Fig. 2B). In addition, DFF45 protein level and PARP cleavage was also decreased in UV-irradiated HR18 cells (Fig. 2B). We also observed that cisplatin-resistant HeLa cells HR6, which expressed a low level of DDB2 [22], also showed increase UV resistance following overexpression of DDB2 (data not shown). These results support the notion that DDB2 protects against UV-induced apoptosis in a caspase-8 and/or caspase-9-dependent manner.

### Overexpression of DDB2 increases cFLIP level and resistance to UV

**Figure 3:**
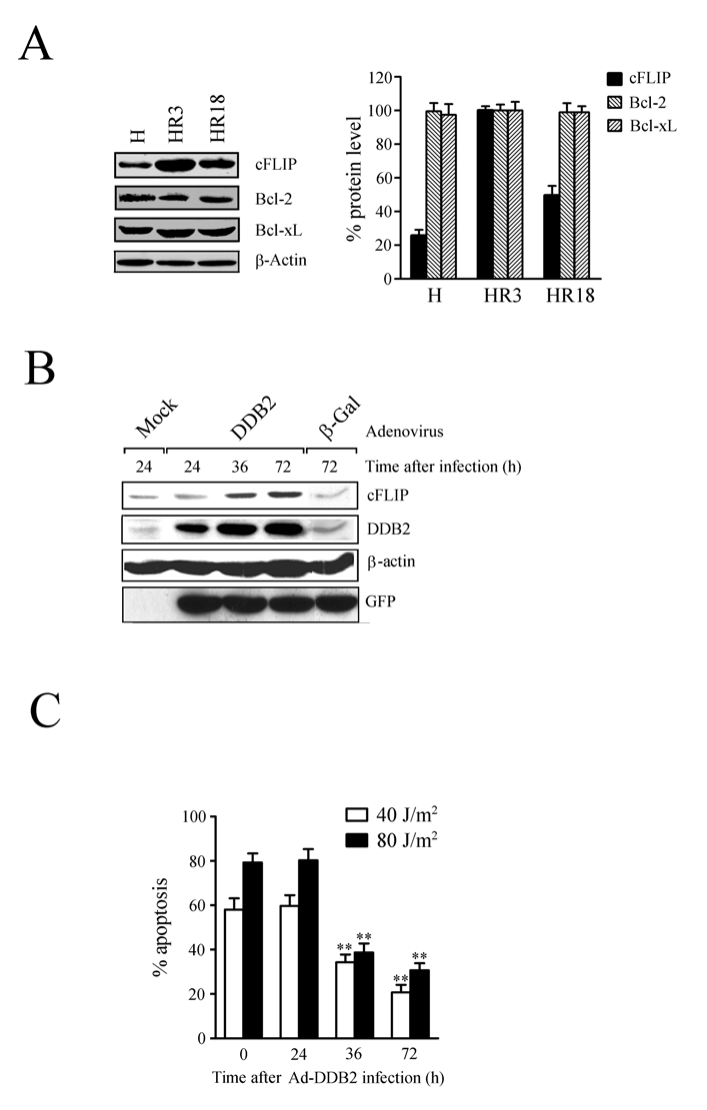
**Stimulation of cFLIP expression and attenuation of UV sensitivity by forced expression of DDB2**. (A) Overexpression of cFLIP in HR3 cells. The plotted values represent means ± S.D. of three experiments (right panel). (B) Stimulation of cFLIP expression by forced expression of DDB2. Whole cell extracts of HR18 cells, treated as indicated, were subjected to immunoblot analysis with specific antibodies. (C) Cell sensitivity to UV after Ad-DDB2 virus infection. The plotted values represent means ± S.D. of three experiments. ** Significant difference against control (*p *< 0.05).

The protective effect of DDB2 against UV irradiation may be associated with various regulators of apoptosis. To assess this possibility, we examined the level of cFLIP, Bcl-2, and Bcl-xL in UV-resistant cells. The level of cFLIP protein was increased in HR3 cells compared to control HeLa cells (Fig. 3A). On the other hand, HR18 cells, which express a low level of DDB2, showed low cFLIP compared to control cells (Fig. 3A). Notably, we found that overexpression of DDB2 using an adenovirus system increased the level of cFLIP in HR18 cells (Fig. 3B). In this case, increased expression of DDB2 was noticed 24 hrs following virus infection, whereas the level of cFLIP increased only 36 hrs following virus infection (Fig. 3B). Overexpression of control Gal did not influence the level of DDB2 or cFLIP compared to mock-treated cells (Fig. 3B). The stimulation of cFLIP following overexpression of DDB2 was also detected in HeLa cells (data not shown). To verify whether DDB2 could enhance resistance to UV, we overexpressed DDB2 in HR18 cells and monitored apoptosis following UV irradiation. Overexpression of DDB2 was shown to protect against UV irradiation in a time-dependent manner (Fig. 3C). UV resistance correlated with the level of cFLIP protein in this case since UV resistance was maximum at 36 and 72 hours following virus infection (Figs. 3B and 3C). These results indicate that UV resistance may be associated with increased levels of DDB2 and cFLIP.

We also monitored the mRNA levels of DDB2 and cFLIP in these cells by using quantitative PCR (Table 1). The relative level of endogenous DDB2 mRNA in HeLa, HR3, and HR18 cells were 1, 1.3, and 0.9, respectively. On the other hand, the level of cFLIP mRNA in HeLa, HR3, and HR18 was respectively 1, 5, and 2.6. Following overexpression of DDB2, HR18 cells displayed a 22-fold increase of DDB2 mRNA and a 3.4-fold increase of cFLIP. In comparison, HR18 cells that overexpressed β-Gal showed a more modest increase of DDB2 mRNA (1.78 fold) and cFLIP (1.9 fold), indicating that virus infection had a low effect on these cells. From these results, we can see that the level of DDB2 and cFLIP is increased in cisplatin-resistant cells, and that the level of these two proteins appears to correlate with resistance to UV.

**Table 1:** Induction of endogenous cFLIP mRNA levels in cells following Ad-DDB2 infection.

	Fold increase of mRNA^a^	
	
Cell/Adv infection	DDB2	cFLIP
HeLa	1.006 ± 0.181	1.006 ± 0.167
HR3	1.302 ± 0.025	5.087 ± 0.298
HR18	0.909 ± 0.088	2.612 ± 0.010
HR18/Ad-β-Gal	1.780 ± 0.314	1.906 ± 0.220
HR18/Ad-DDB2	22.851 ± 0.115	3.446 ± 0.123^b^

^a^The numbers indicate mean ± standard deviation of three experiments.

Those samples were examined 60 h after virus infection.

^b^Significant difference to HR18/Ad-β-Gal, p < 0.01.

### Low upregulation of cFLIP promoter activity by overexpression of DDB2

**Figure 4:**
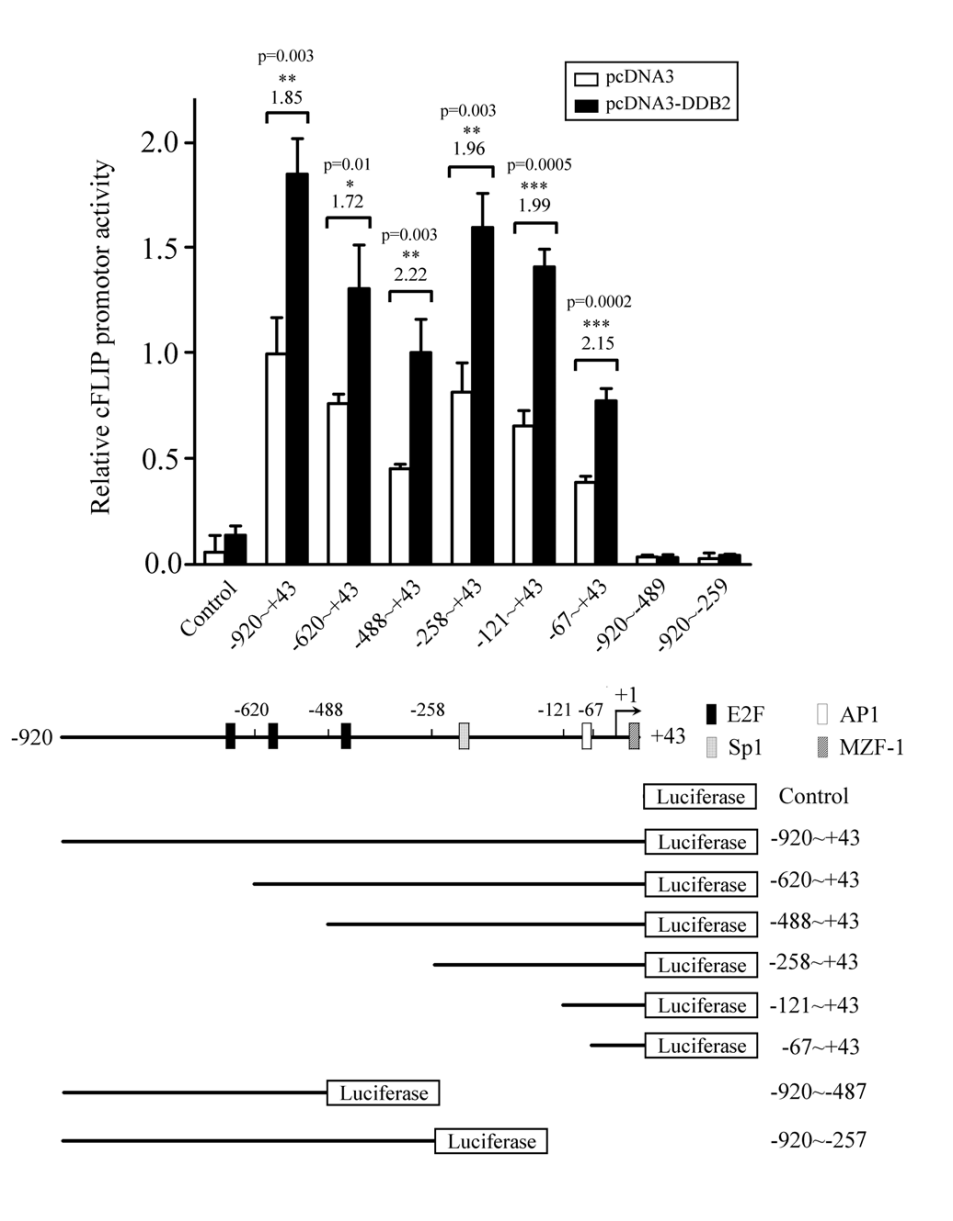
**Upregulation of cFLIP promoter activity by forced expression of DDB2**. HEK293 cells were co-transfected with cFLIP reporter together with either control vector (pcDNA3) or DDB2-expressing vector (pcDNA3-DDB2). After 24 hrs, the luciferase activity was measured. The plotted values represent means ± S.D. from three independent transfections. The schematic presentation of full-length cFLIP promoter (-920/+43) and its deletion mutants are indicated below. Putative cis-elements are also indicated at positions relative to the transcription initiation site (+1). The construct number at the top indicates the length of the tested promoter region upstream of the putative transcription initiation site (designated by the bent arrow at +1). Luciferase activity is shown relative to the full-length cFLIP promoter (-920/+43). Significant difference to the control for each promoter is indicated.

To examine whether DDB2 may upregulate cFLIP gene by activating its transcriptional, a cFLIP promoter (-920/+43, setting the transcription initiation site as +1), which had been fused to a luciferase cDNA as a reporter gene, was co-transfected with a plasmid expressing DDB2 (pcDNA3-DDB2) in HEK293 cells. The cFLIP promoter contains several potential cis-acting elements for transactivators, including E2F (Fig. 4, bottom panel. A series of deletion mutants were constructed as indicated (Fig. 4, bottom panel). Transient expression analysis in HEK293 cells indicated the presence of cFLIP core promoters located 920 bp upstream of the putative transcription initiation sites. Deletion of these elements reduced basal promoter activity (Fig. 4, top panel, open bars). The core promoters contain multiple active E2F sites, followed by a site at -488 to -258, which represents a critical determinant of negative regulation for this promoter activity. In addition, Sp1 and AP1 sites located at -158 to -67 may be essential transcription elements. Overexpression of DDB2 induced nearly a two-fold increase of FLIP promoter activity in HEK293 cells (Fig. 4, top panel). Notably, all the 5'-deletion mutants displayed similar promoter activities as the full-length promoters (Fig. 4, -920/+43). The promoter activity was undetected in the 3'-deletion mutants (-920/-487 and -920/-258) where sequences spanning the transcriptional initiation site were deleted. These results suggest that DDB2 slightly enhances cFLIP promoter activity, and that the trans-activation effect may involve multiple transcription factors.

### Knockdown of cFLIP using antisense oligonucleotides decreases the anti-apoptotic effects of DDB2 against UV irradiation

**Figure 5:**
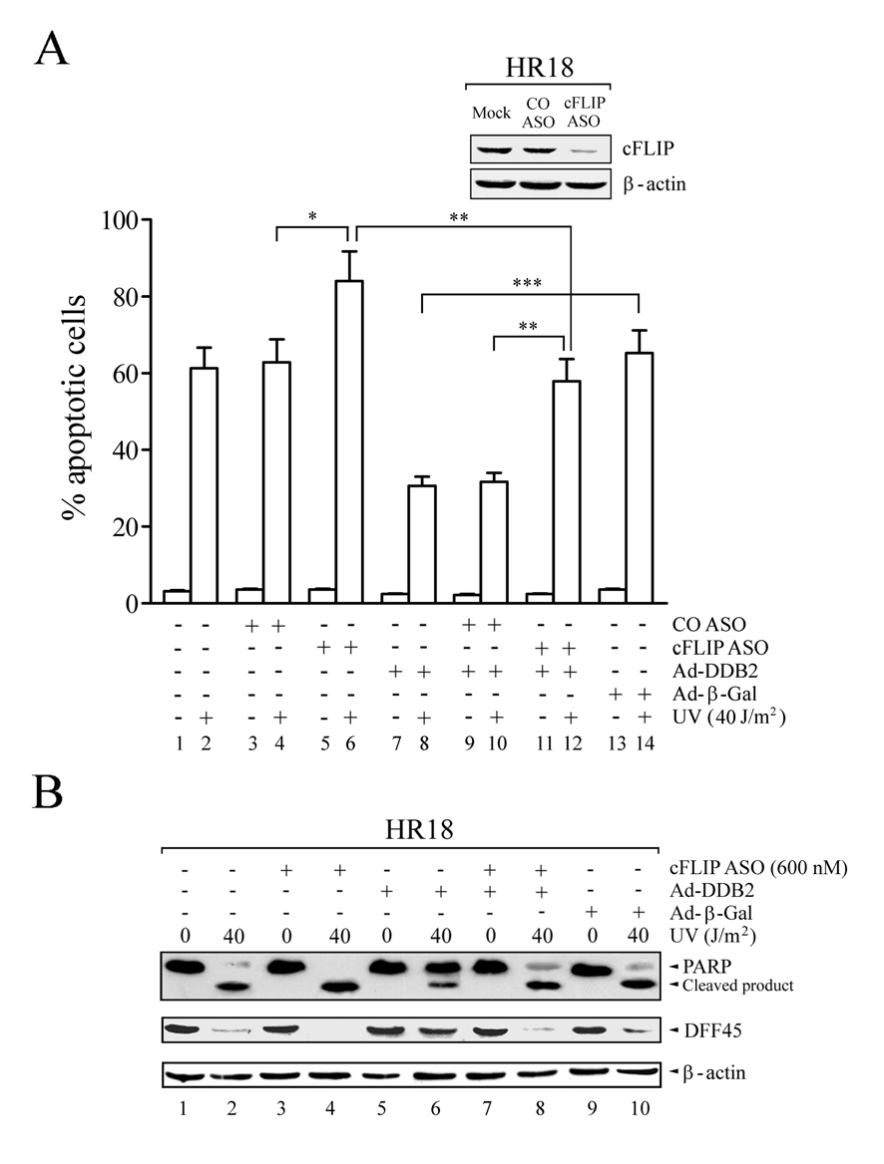
**Resistance to UV in HR18 cells depends on DDB2 and cFLIP**. (A) Attenuation of DDB2 protection against UV-induced apoptosis by knockdown of cFLIP using antisense oligonucleotides. HR18 cells were treated as described in the *Materials and methods*. The plotted values represent means ± S.D. of experiments performed in triplicates. Inset: immunoblot with specific antibodies. (B) Attenuation of UV-induced caspase activity by forced expression of DDB2, and resensitization by cFLIP antisense oligonucleotides.

The data presented above suggest that cFLIP mediates the protective effect of DDB2 against UV-induced apoptosis. To test this possibility more directly, we used cFLIP antisense oligonucleotides (ASO) to decrease the level of this protein in HR18 cells. The level of cFLIP was efficiently decreased by treatment with 600 nM of cFLIP antisense ASO, whereas control ASO ("CO ASO") did not affect this protein (Fig. 5A, insert). As shown in Figure 5A, cFLIP antisense markedly sensitized HR18 cells to apoptosis following UV irradiation. Notably, overexpression of DDB2 was shown to partially protect HR18 cells against apoptosis induced by UV (Fig. 5A). In contrast, forced expression of control β-Gal did not exhibit any protective effect (Fig. 5A). cFLIP ASO attenuated the protective effects of DDB2 overexpression against UV-induced apoptosis (Fig. 5A).

Overexpression of DDB2 also decreased UV-induced cleavage of both DFF and PARP in HR18 cells (Fig. 5B). In contrast, forced expression of control β-Gal did not exhibit any protection effect on the cleavage of either DFF or PARP (Fig. 5B). cFLIP ASO decreased the protective effect of DDB2 overexpression against UV-induced cleavage of DFF and PARP (Fig. 5B). However, the apoptosis level of HR18 cells that overexpressed DDB2, and which were treated with cFLIP ASO, was still lower than that of control cFLIP-suppressed cells (Fig. 5B). In this case, the level of apoptosis was more significant in DDB2-expressing cells (p < 0.01) compared to control cells (p < 0.05) (Fig. 5). This observation suggests that the activation of cFLIP by DDB2 may play a more protective role against UV than endogenous cFLIP. These results indicate that DDB2 protection against UV-induced apoptosis may proceed via a pathway regulated by cFLIP.

### Overexpression of DDB2 in cells lacking cFLIP does not offer a protective effect against UV irradiation

**Figure 6:**
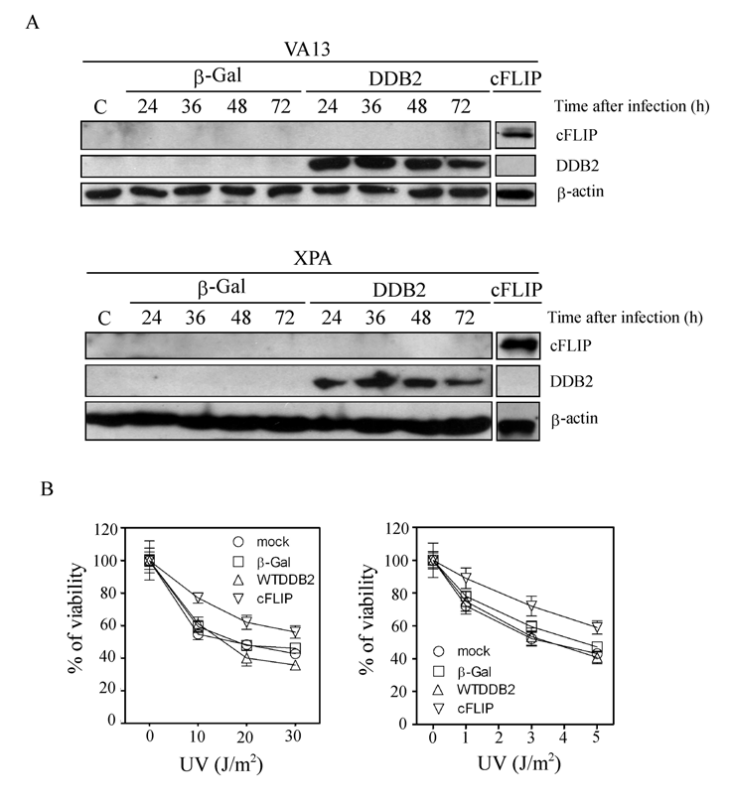
**Lack of attenuation of UV sensitivity by forced expression of DDB2 in cFLIP-lacking cells**. (A) Lack of stimulation of cFLIP expression by forced expression of DDB2 in human VA13 and XP-A cells. Whole cell extracts of infected cells were compared. Cell extracts of cFLIP-transfected cells are also shown as cFLIP protein indicator. (B): Cell sensitivity to UV treatment after Ad-DDB2 virus infection in human VA13 and XP-A cells. The plotted values represent means ± S.D. of three experiments.

We also used human VA13 and XP-A cells which express low level of cFLIP. Surprisingly, these cells did not display upregulation of cFLIP following DDB2 overexpression (Fig. 6A). Notably, sensitivity to UV was not affected by overexpression of DDB2 in VA13 and XP-A cells (Fig. 6B). Cell extracts of cFLIP cDNA transfected cells are included as cFLIP protein marker. Besides, we observed that overexpression of cFLIP substantially enhanced cell viability in both VA13 and XP-A cells (Fig. 6B). as well as in HEK293 cells (data not shown).

### Protection against UV toxicity by DDB2 in Drosophila

**Figure 7:**
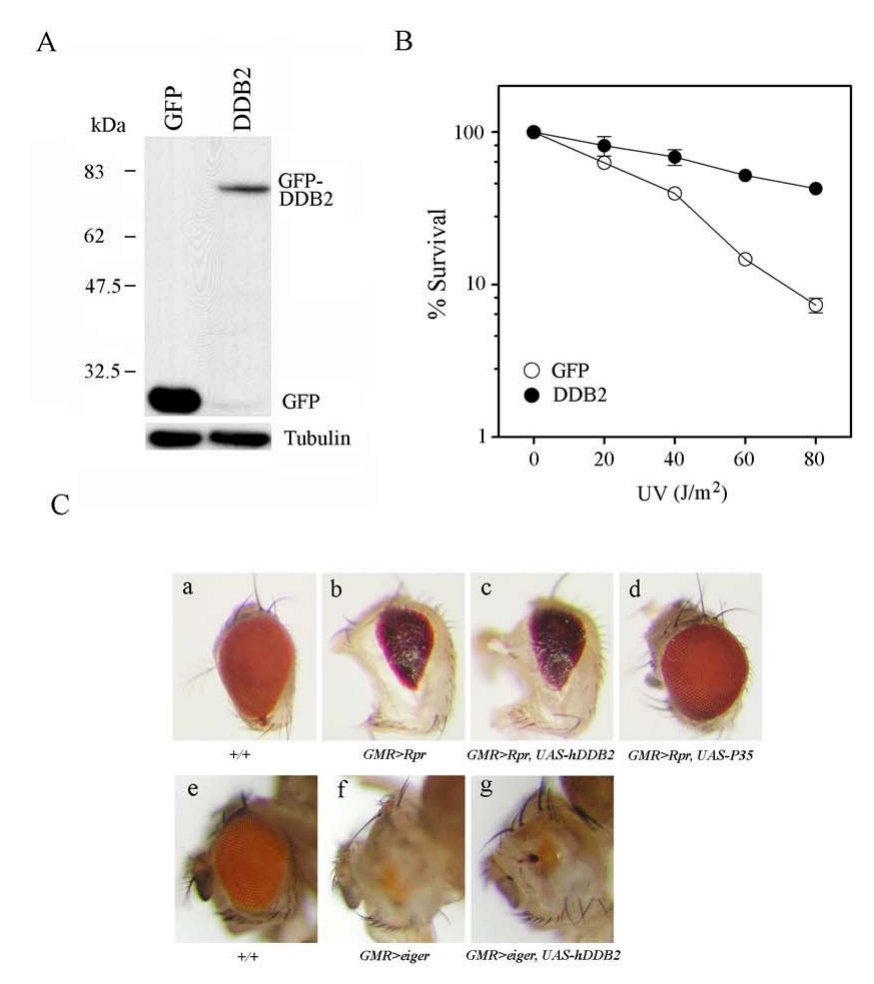
**Protection against UV toxicity by DDB2 in *Drosophila***. (A) Expression of hDDB2-GFP in *Drosophila*. Protein extracts from GFP and GFP-hDDB2 expressing embryos were immunoblotted with anti-GFP antibodies. (B) Protection effect of hDDB2 against UV irradiation in fly larvae. GFP or hDDB2 overexpressing larvae were collected and then irradiated with UV (0-100 J/m^2^). Four days later, surviving adults were counted and the survival rate was calculated. (n>4000). (C) Lack of inhibition of Reaper- or Eiger-induced apoptosis by hDDB2 over-expression. Eye morphology of wild-type and Reaper/eiger-overexpressing flies were examined. To analyze the effect of hDDB2, GMRGal4 was used to drive the expression of Reaper (*GMR>Rpr*) or Eiger (*GMR>eiger*) simultaneously with hDDB2 (*GMR>Rpr, UAS-hDDB2 *and *GMR>eiger, UAS-hDDB2*, respectively). Panel a-d: Reaper-induced apoptosis, resulting in small eyes, was not rescued by DDB2 overexpression. Panel a, Eye morphology of wild-type OreR fly; Panel b, Eye morphology of Rpr overexpressing fly; Panel c, Eye morphology of Rpr and DDB2 overexpressing fly; Panel d, Eye morphology of Rpr and P35 overexpressing fly. Panel e~g: Eiger-induced apoptosis not rescued by DDB2 overexpression. Panel e, Eye morphology of wild-type OreR fly; Panel f, Eye morphology of eiger overexpressing fly; Panel g, Eye morphology of eiger and DDB2 overexpressing fly.

Earlier, we and others found that overexpression of DDB2 enhances nuclear excision repair in both hamster [16,17] and human cells [18,19]. To explore whether the protective role of DDB2 is conserved in other living organisms, we expressed human DDB2 cDNA in the fruit fly *Drosophila*, and exposed the resulting flies to UV irradiation. DDB2-GFP fusion construct and GFP control construct were expressed in *Drosophila *with nuclear fluorescence signals in the salivary gland of the third instar larvae (data not shown). To detect protein expression, we isolated proteins from embryos or third instar larvae after a heat-shock induction of 2 hrs. The DDB2-GFP protein were detected by Western blot using mouse anti-GFP antibody. In this case, the molecular weight of DDB2-GFP is 75 kDa and that of GFP is 27 kDa. UV-induced toxicity was considerably suppressed in flies that overexpressed DDB2 compared to control flies that overexpressed GFP (Fig. 7B).

We also verified whether DDB2 could prevent apoptosis induced by the pro-apoptotic genes *Rpr *or *eiger *in fruit flies. We first observed that flies that overexpressed either *Rpr *or *eiger *showed apoptosis in the eyes as shown by the reduced eye size compared to control flies (Fig. 7C, panels a and e vs. panels b and f, respectively). On the other hand, overexpression of DDB2 failed to rescue this apoptotic effect (Fig. 7, panels c and g). In control experiments, apoptosis induced by activated *Rpr *could be mostly rescued by overexpression of p35 (Fig. 7C, panel b vs. panel d). Even severe apoptosis in the eye was resulted by activated *eiger *(Fig. 7C, compare panel e and panel f). These results suggest that overexpression of DDB2 may protect against UV toxicity, but that DDB2 is unable to rescue activated apoptosis in *Drosophila*.

## Discussion

In the present study, we demonstrated that DDB2 increased resistance to UV irradiation in a cFLIP-dependent manner in cisplatin-selected HeLa cells. The marked decrease of apoptosis following overexpression of DDB2 may partially explain why cisplatin-selected cells are cross-resistant to UV [21,22] and TNF treatments [26,27]. Although cisplatin-induced apoptosis can be mediated by the Fas signaling pathway [27,28], this pathway involves the mitochondria and the action of caspase-9 [29]. However, attenuation of intracellular DDB2 levels in HR3 cells did not affect apoptosis induced by either cisplatin or mitomycin C, which potentially stimulate mitochondrial death signals [22]. These observations suggest that DDB2 may not be involved in regulating mitochondrial death pathway. Therefore, the increase of DDB2 and cFLIP expression in cisplatin-resistant cells may explain the cross-resistance to UV in these cells, but not resistance to cisplatin.

Overexpression of DDB2 has also been shown to promote global genomic repair in hamster cells [16,17]. Notably, forced expression of mutant DDB2 (DDB2-82TO), which is defective in DDB1 interaction and damage recognition [30], also protects cells against UV-induced apoptosis in HeLa cells [22]. These results strongly suggest that the regulation of UV-induced apoptosis by DDB2 may be independent of DNA repair in these cells. Thus, DDB2 as a DNA repair protein also has a role in regulating cell response to agents that activate cell surface death signals such as UV and TNF. Importantly, the protection effect of DDB2 against UV was only detected in cells whose cFLIP levels are accumulated at high levels (Fig. 2). This protection effect was decreased when cFLIP levels became low or were attenuated by ASO (see Figs. 3 and 5). However, overexpression of DDB2 in hamster cells may or may not exhibit protection against UV [16,17]. These divergent observations may be due to different treatments during which cellular level of cFLIP likely has a critical role in UV resistance. In addition, forced expression of DDB2 in cFLIP-lacking cells (VA13 and XP-A) did not induce cFLIP accumulation, or protection against UV (Fig. 6), indicating that the DDB2-cFLIP pathway responsible for the protective effect against UV-induced apoptosis is probably not evolutionarily conserved.

DDB2 transcription can be stimulated by E2F1, which does not require p53, but can be potentiated by this protein in primary mouse hepatocytes [31]. Moreover, microarray analysis has demonstrated that FLIP is one of the E2F1-regulated genes in Saos-2 cells [32]. These findings suggest that E2F1 may also play a role in upregulating cFLIP, thereby exerting an apoptotic resistance by inhibiting DISC formation [33] in the acquisition of UV resistance. Consistent with this idea, we observed that E2F1 accumulated in UV-resistant HeLa cells. However, E2F1 has also been implicated in causing apoptosis [34]. Additional overexpression of E2F1 does not increase endogenous cFLIP expression more than overexpression of DDB2 alone (data not shown). Thus, the increased E2F1 level in the resistant cells is not enough to support apoptotic resistance mediated by DDB2-cFLIP. Although induction of cFLIP by DDB2 is required for protecting cells from UV-induced apoptosis, at least in HeLa cells, we could not exclude the possible involvement of other genes expression for DDB2-induced cross-resistance. The expression of DDB2 is also transcriptionally regulated by p53 in cell-type dependent manner [35]. Since HeLa cells express lower levels of p53 due to infection with human papillomavirus, continuous exposure of cells to cisplatin during selection for resistance may activate p53 and increase DDB2 [35], thereby upregulating cFLIP levels and providing an opportunity for the cells to escape UV-induced apoptosis. An apoptotic threshold significantly regulated by p53-Bcl2 connection has been proposed [36]. In this model, p53-dependent signals, like the induction of Bax and direct inhibition of Bcl2, may synergize with p53-independent signals including the induction of Bim to antagonize Bcl2 function and promote apoptosis. This model may explain the chemotherapeutic response of cancer cells as most of the DNA modifying anti-cancer drugs induce mitochondria death pathway. Our findings herein, together with others, suggest that DDB2-cFLIP or p53-dependent DDB2-cFLIP expression, accounts for an additional route in cell resistance for agents that preferentially evoke cell surface death pathway in specific cell type.

We have previously demonstrated that overexpression of DDB2 could potentiate DNA repair and protect against UV toxicity in human HeLa and hamster V79 cell lines [16,22]. Similarly, overexpression of DDB2 protects against UV toxicity in *Drosophila*, suggesting that DDB2 may exert its protective activity both *in vitro *and *in vivo*. Other authors have also shown that DDB2 could enhance global genomic repair and suppress UV-induced mutagenesis in rodent cells [17]. The effects of DDB2 on DNA repair was further supported by recent studies. For example, overexpression of DDB2 potentiated nuclear excision repair in mouse embryonic fibroblasts that were irradiated with low doses of UV as shown by accumulation of DDB1 in the nucleus, degradation of p53, and low level of p21^Waf1/Cip1^, which is believed to be an inhibitor of repair synthesis [37]. In addition, knockdown of DDB2 in MCF-7 cells caused a decrease of cancer cell growth and colony formation. Inversely, introduction of the DDB2 gene into MDA-MB231 (low DDB2) cells stimulated growth and colony formation [38]. DDB2 may play a role in potentiation of MCF-7 cell growth by exerting a negative regulation of the *sod2 *gene [39]. Hence, DDB2 also plays an important role in the positive regulation of cell growth. In most cases, DDB2 overexpression only partially reverses induced apoptosis, suggesting that severe damage in cells may override the protective function of DDB2. Surprisingly, however, overexpression of DDB2 is unable to rescue activated apoptosis (induced by *rpr *or *eiger*) in *Drosophila*. As such, *Drosophila *with less potent apoptosis design is needed to re-examine the effect of DDB2 in regulation of apoptosis in flies. Recently, the potentiation or lacking effects of DDB2 on DNA damage-induced apoptosis has also been reported in different cell types [40-42]. It is concluded that different genetic make-up between cell types may play an important role in the regulation of DDB2-mediated cell response to UV stress.

## Conclusion

Our results show that DDB2 protects cells against UV irradiation via the action of cFLIP, which mediates an anti-apoptosis response following irradiation. Ectopic expression of human DDB2 in the fruit fly *Drosophila *also inhibited UV-induced fly death but it failed to rescue apoptosis activated by either *Reaper *or *eiger *gene. The protective role of DDB2 against UV stress may be conserved in various living organisms, whereas cFLIP expression may be one of the many mechanisms in mediating protective DDB2 during the acquisition of apoptotic resistance.

## Abbreviations

ASO: antisense oligonucleotides; CDDP: cisplatin; cFLIP: FLICE inhibitory proteins; DDB2: UV-DNA damage binding protein 2; DFF: DNA fragmentation factor; β-Gal: β-Galactosidase; GAPDH: glyceraldehyde 3-phosphate dehydrogenase; GFP: green fluorescence protein; MTT: 3-(4,5-dimethylthiazol-2-yl)-2,5-diphenyltetrazolium bromide; PARP: Poly-ADP ribose polymerase; PCR: polymerase chain reaction; SDS-PAGE: sodium dodecyl sulfate-polyacrylamide gel electrophoresis; UV: ultraviolet radiation. XP-A: xeroderma pigmentosum group A.

## Competing interests

The authors declare that they have no competing interests.

## Authors' contributions

NKS, CLS, and CCKC conceived and designed the experiments. NKS, CLS, and CHL performed the experiments. NKS, CLS, LMP, and CCKC analyzed the data. CCKC wrote the paper. All authors have read and approved the final manuscript.
